# The prevalence, penetrance, and expressivity of etiologic *IRF6* variants in orofacial clefts patients from sub‐Saharan Africa

**DOI:** 10.1002/mgg3.273

**Published:** 2017-01-12

**Authors:** Lord Jephthah Joojo Gowans, Tamara D. Busch, Peter A. Mossey, Mekonen A. Eshete, Wasiu L. Adeyemo, Babatunde Aregbesola, Peter Donkor, Fareed K. N. Arthur, Pius Agbenorku, James Olutayo, Peter Twumasi, Rahman Braimah, Alexander A. Oti, Gyikua Plange‐Rhule, Solomon Obiri‐Yeboah, Fikre Abate, Paa E. Hoyte‐Williams, Taye Hailu, Jeffrey C. Murray, Azeez Butali

**Affiliations:** ^1^ Department of Biochemistry and BiotechnologyKwame Nkrumah University of Science and Technology (KNUST)KumasiGhana; ^2^ Cleft ClinicKomfo Anokye Teaching HospitalKumasiGhana; ^3^ Department of PaediatricsUniversity of IowaIowa CityIowa; ^4^ Department of Oral PathologyRadiology and MedicineUniversity of IowaIowa CityIowa; ^5^ Department of OrthodonticsUniversity of DundeeDundeeScotland; ^6^ Addis Ababa UniversityAddis AbabaEthiopia; ^7^ College of MedicineUniversity of LagosLagosNigeria; ^8^ Obafemi Awolowo University Teaching HospitalIle‐IfeNigeria; ^9^ Department of SurgerySchool of Medical SciencesKNUSTKumasiGhana

**Keywords:** Craniofacial genetics, expressivity, penetrance, population genetics, rare variants, Van der Woude syndrome

## Abstract

**Background:**

Orofacial clefts are congenital malformations of the orofacial region, with a global incidence of one per 700 live births. Interferon Regulatory Factor 6 (*IRF6*) (OMIM:607199) gene has been associated with the etiology of both syndromic and nonsyndromic orofacial clefts. The aim of this study was to show evidence of potentially pathogenic variants in *IRF6* in orofacial clefts cohorts from Africa.

**Methods:**

We carried out Sanger Sequencing on DNA from 184 patients with nonsyndromic orofacial clefts and 80 individuals with multiple congenital anomalies that presented with orofacial clefts. We sequenced all the nine exons of *IRF6* as well as the 5′ and 3′ untranslated regions. In our analyses pipeline, we used various bioinformatics tools to detect and describe the potentially etiologic variants.

**Results:**

We observed that potentially etiologic exonic and splice site variants were nonrandomly distributed among the nine exons of *IRF6*, with 92% of these variants occurring in exons 4 and 7. Novel variants were also observed in both nonsyndromic orofacial clefts (p.Glu69Lys, p.Asn185Thr, c.175‐2A>C and c.1060+26C>T) and multiple congenital anomalies (p.Gly65Val, p.Lys320Asn and c.379+1G>T) patients. Our data also show evidence of compound heterozygotes that may modify phenotypes that emanate from *IRF6* variants.

**Conclusions:**

This study demonstrates that exons 4 and 7 of *IRF6* are mutational ‘hotspots’ in our cohort and that *IRF6* mutants‐induced orofacial clefts may be prevalent in the Africa population, however, with variable penetrance and expressivity. These observations are relevant for detection of high‐risk families as well as genetic counseling. In conclusion, we have shown that there may be a need to combine both molecular and clinical evidence in the grouping of orofacial clefts into syndromic and nonsyndromic forms.

## Introduction

Orofacial clefts (OFCs) are congenital craniofacial abnormalities with a global prevalence of one per 700 live births (Mossey and Modell 2012). OFCs may be syndromic or nonsyndromic, with the syndromic forms presenting with other congenital malformations such as lip pits, heart defects, hexadactyly, etc. Nonsyndromic OFCs (NSOFCs) may present as nonsyndromic cleft lip only (NSCL), nonsyndromic cleft lip and palate (NSCLP) and nonsyndromic cleft palate only, NSCPO (Dixon et al. 2011). In the Online Mendelian Inheritance in Man database (OMIM), over 300 different syndromes present with clefts (www.omim.org). Van der Woude Syndrome (VWS, OMIM:119300) and Popliteal Pterygium Syndrome (PPS, OMIM:119500) are allelic syndromes that are characterized by clefts and other anomalies. These syndromes emanate mainly from etiologic variants in *IRF6* (OMIM:607199) gene (Kondo et al. 2002), though *GRHL3* (OMIM:608317) (Peyrard‐Janvid et al. 2014) has been shown to be mutated in about 5% of VWS patients that lack etiologic variants in *IRF6*. VWS is the most common syndromic form of cleft, accounting for about 2% of all clefts and increases the recurrence risk about threefold (Ghassibe et al. 2004). Nonpathogenic common variants (Zucchero et al. 2004) and functionally validated single‐nucleotide polymorphism (SNP) in *AP‐2α* enhancer element (Rahimov et al. 2008) in and near *IRF6* have also been associated with nonsyndromic cleft lip with or without palate (NSCL/P).

Inasmuch as over 300 etiologic variants in *IRF6* have been reported worldwide in patients with VWS and PPS, these variants are nonrandomly distributed among the seven coding exons of the gene (Kondo et al. 2002; Ferreira de Lima et al. 2009; Leslie et al. 2013; Butali et al. 2014a). These variants are usually concentrated in the highly conserved DNA‐binding domain (exons 3 and 4) and less conserved transactivation domain (exons 7 and 9) (Ferreira de Lima et al. 2009). Recent sequence analyses of these four exons in VWS and PPS patients from Africa confirmed that these four exons are mutational “hot‐spots” in *IRF6* (Butali et al. 2014a). The study, however, involved only two African populations, Ethiopia and Nigeria. This study mainly sought to detect etiologic *IRF6* variants in both NSOFC and multiple congenital anomalies (MCA) patients from Ghana, an unstudied population. We also directly sequenced *PAX7* (OMIM: 167410) in our NSOFC Ghanaian cohort. Moreover, MCA patients from Ethiopia and Nigeria were also included in the genetic analyses that involved direct DNA sequence analyses of all the 9 exons of *IRF6*. MCA patients were screened for *IRF6* pathogenic variants for two reasons. Firstly, to detect etiologic variants in classical VWS patients as well as patients with spectrum of phenotypes that have been reported in PPS patients. Secondly, all syndromes were included in the MCA cohort so as to ascertain whether *IRF6* etiologic variants modify the phenotypes of these syndromes.

## Materials and Methods

### Ethical compliance

All sample and data collection at various study sites were approved by the local Institutional Review Boards: College of Health Sciences, Kwame Nkrumah University of Science and Technology (KNUST), Ghana – CHRPE/AP/217/13, College of Medicine, University of Lagos, Nigeria – ADM/DCST/HREC/APP/1374 and College of Health Sciences, Addis Ababa University, Ethiopia ‐ 3.10/027/2015. Before sample collection, written informed consent was obtained from all participants.

### Subjects

We selected 184 NSOFC (60 NSCL, 71 NSCLP and 53 NSCPO) and 54 MCA patients from indigenous Ghanaian patients with OFC that were encountered by the Cleft‐Craniofacial Team of Komfo Anokye Teaching Hospital (KATH), Kumasi, Ghana. Moreover, to increase the sample size for MCA patients, we added six and 20 MCA patients from Ethiopia and Nigeria, respectively, to the study cohort. The Ethiopian patients were recruited from Yekatit 12 Hospital, Addis Ababa, whereas the Nigerian patients were recruited from University of Lagos Teaching Hospital and Obafemi Awolowo University Teaching Hospital. In all, 80 MCA patients formed the study cohort.

**Figure 1:**
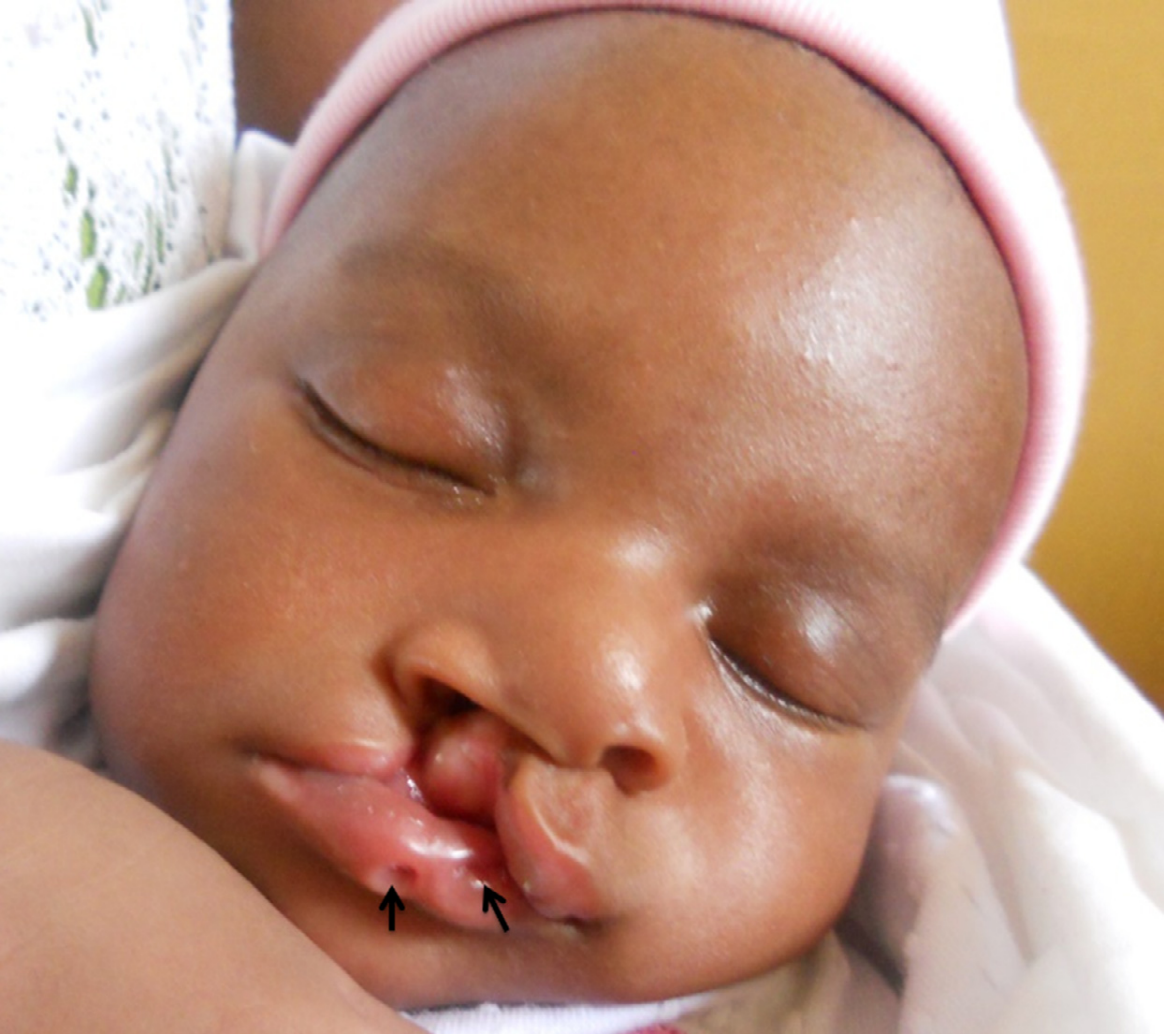
A patient with Van der Woude Syndrome that presented with CLP and bilateral lip pits (indicated by arrow heads).

At the time of sample collection and clinical examination, none of the NSOFC patients showed any other clinical abnormalities in addition to the OFCs. All OFC patients that presented with any congenital deformity in addition to OFC were classified as having MCAs. These patients included those with known syndromes like VWS, Pierre Robin Sequence (PRS), Apert Syndrome, Kabuki Syndrome, Moebius Syndrome, Stickler's syndrome, etc. Other patients with OFC also presented with club foot only, hexadactyly, heart defects, microphthalmia, anophthalmia, hypospadias, ankyloglossia, imperforate anus, etc. (Fig. 1; Table S1).

### DNA processing, PCR and electrophoresis

Saliva or cheek swab samples were collected from all participants, using the Oragene DNA Collection Kits (http://www.dnagenotek.com). We extracted DNA from all samples using the Oragene Saliva processing protocol. We then quantified the DNA, using Quibit Assay that employed Quibit 2.0 Fluorometer (http://www.invitrogen.com/site/us/en/home/brands/Product-Brand/Qubit.html), followed by XY‐Genotyping to validate the sexes of the samples. Primer sequences that were used to amplify exons 1 to 9 of *IRF6* (RefSeq NM_006147.3) and *PAX7* (RefSeq NM_001135254.1) have been published elsewhere (Ferreira de Lima et al. 2009; Butali et al. 2014a,b). All DNA processing protocols, PCR conditions and electrophoretic procedure are available at the Murray laboratory website (http://genetics.uiowa.edu/protocols.php).

### Analyses of DNA sequence results

Amplified DNA products were sequenced at Functional Biosciences in Madison, Wisconsin (http://order.functionalbio.com/seq/index), using an ABI 3730XL DNA Sequencer (http://www.appliedbiosystems.com/absite/us/en/home.html) that employed Sanger Sequencing Technology. Chromatograms were then transferred to a Unix workstation, base‐called with PHRED (http://www.phrap.org/phredphrapconsed.html, v.0.961028), assembled with PHRAP (http://www.phrap.org/, v.0.960731), scanned by POLYPHRED (http://droog.gs.washington.edu/polyphred/, v. 0.970312) and viewed with CONSED programme (http://www.phrap.org/consed/consed.html, v. 4.0).

The genomic location of each variant revealed by CONSED was ascertained using the “Blat” function of UCSC Genome Browser (https://genome.ucsc.edu/). The functional effect of a coding variant on protein was predicted, using Polyphen‐2 (http://genetics.bwh.harvard.edu/pph2/) and Sift (http://sift.jcvi.org/). Simulation of mutant protein structure was carried out using HOPE (http://www.cmbi.ru.nl/hope). Effect of a variant on mRNA splicing was ascertained using Human Splicing Finder 3.0 (http://www.umd.be/HSF3/). Furthermore, effect of a variant on a regulatory region was predicted, using RegulomeDB (http://regulomedb.org/).

The Minor Allele Frequencies or novelty of a variant was ascertained by comparing it to variants in 1000 Genomes (http://browser.1000genomes.org/index.html), Exome Variant Server (http://evs.gs.washington.edu/EVS/), dbSNP (www.ncbi.nlm.nih.gov/SNP/) and ExAC Browser (http://exac.broadinstitute.org/). Variants were classified as “novel” if they have never been reported in any of these databases or literature. Upon detection of a variant of interest in proband, samples from other relatives as well as those of the probands, were then sequenced in the reverse direction to confirm the variant and also to ascertain whether a variant was de novo or segregated in a particular family. All novel coding variants have been deposited in a cleft lip and palate mutation database called LOVD (http://www.icp.ucl.ac.be/vikkula/CLPdb). The accession numbers are: c.194G>T, *IRF6*_00051; c.205G>A, *IRF6*_00052; c.554A>C, *IRF6*_00053 and c.960G>C, *IRF6*_00054.

## Results

**Figure 2:**
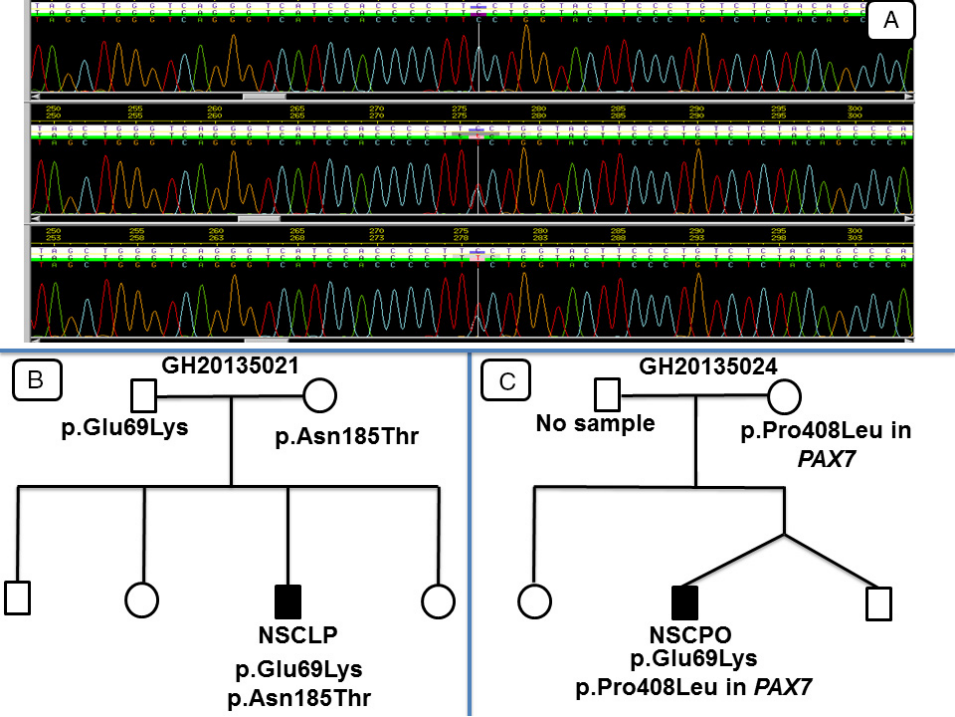
DNA Sequence chromatograms and pedigrees for p.Glu69Lys variant. (A): top chromatogram is from an unaffected individual, whereas the middle and bottom chromatograms are from two patients with p.Glu69Lys variant, (B): a case with *IRF6* (RefSeq NM_006147.3) compound heterozygote, (C): a case with *IRF6‐PAX7* (RefSeq NM_001135254.1) compound heterozygote.

After the various analyses pipelines stipulated in the methodology, we observed 14 different rare and potentially pathogenic variants in *IRF6* in both syndromic and nonsyndromic cleft patients (Table 1, Fig. 2). Seven of these variants were novel. In silico analyses were based on Ensemble transcript number ENST00000367021 for *IRF6*. It must be noted that p.Asn185Thr was observed in an individual with p.Glu69Lys variant. Apart from this, all variants stated in the table were observed in different individuals.

**Table 1:** Rare and potentially pathogenic variants observed in *IRF6*

HGVS	HGVp	*a*	*b*	Polyphen‐2	Sift	Human splice finder	RDB	Reference
^*α*^chr1:209,979,529 A>T (rs34743335)	N/A	5	1	N/A	N/A	N/A	*i*	29
c.175‐2A>C	N/A	1	0	N/A	N/A	*c*	N/A	Novel
c.194G>T	p.Gly65Val	0	1	Probably Damaging	Deleterious	N/A	N/A	Novel
c.205G>A	p.Glu69Lys	2	0	Probably Damaging	Deleterious	N/A	N/A	Novel
c.263A>G	p.Asn88Ser	1	0	Probably Damaging	Deleterious	N/A	N/A	14
c.334C>G	p.Gln112Glu	2	0	Benign	Tolerated	N/A	N/A	29
c.379+1G>T	N/A	0	1	N/A	N/A	*d*	N/A	Novel
c.380‐116T>A	N/A	5	2	N/A	N/A	*e*,* f*,* g*	N/A	29
c.554A>C	p.Asn185Thr	1	0	Benign	Tolerated	*h*	N/A	Novel
c.749G>A	p.Arg250Gln	0	1	Probably Damaging	Deleterious	N/A	N/A	14
c.748C>T	p.Arg250X	1	2	N/A	N/A	N/A	N/A	14
c.945G>A	p.Arg315Arg	1	0	Benign	Tolerated	*h*	N/A	30
c.960G>C	p.Lys320Asn	0	1	Probably Damaging	Deleterious	N/A	N/A	Novel
c.1060+26C>T	N/A	1	0	N/A	N/A	N/A	*i*	Novel

*a*: Total number observed in NSCL/P patients, *b*: Total number observed in syndromic clefts, *c*: Alteration of the wild‐type acceptor site – most probably affecting splicing, *d*: Alteration of the wild‐type donor site – most probably affecting splicing, *e*: Activation of an intronic cryptic acceptor site, *f*: Activation of an intronic cryptic donor site, *g*: creation of an intronic ESE site, *h*: Alteration of an exonic ESE site; potential alteration of splicing, *i*: 2b‐Likely to affect binding of *POLR2A*. RDB: RegulomeDB software, *α*: no HGVS available for this variant. Transcript variant 1 of *IRF6* (RefSeq NM_006147.3) based on human genome assembly GRCh37/hg19 of 2009 was sequenced. All novel coding variants have been deposited in a cleft lip and palate mutation database called LOVD (http://www.icp.ucl.ac.be/vikkula/CLPdb). The accession numbers are: c.194G>T, *IRF6*_00051; c.205G>A, *IRF6*_00052; c.554A>C, *IRF6*_00053 and c.960G>C, *IRF6*_00054. ^14^Ferreira de Lima RLL, Hoper SA, Ghassibe M et al. Prevalence and nonrandom distribution of exonic mutations in Interferon Regulatory Factor 6 (*IRF6*) in 307 families with Van der Woude syndrome and 37 families with popliteal pterygium syndrome. Genet Med. 2009: 11(4): 241–247; ^29^ www.ncbi.nlm.nih.gov/SNP/; ^30^ http://exac.broadinstitute.org/.

A VWS patient with bilateral CLP and lip pits had the p.Gly65Val variant; the variant was not in the mother but there was no sample from the father. The novel p.Glu69Lys variant occurred in two NSOFC Ghanaian patients: one with NSCPO and the other with right NSCLP with bifid uvula. This variant segregated in a pedigree, where the variant occurred in both the proband and father, though the father had no overt cleft. In another pedigree, the variant occurred in the proband, but not the only maternal sample available. Both clinically unaffected father and an NSCLP patient had the p.Asn88Ser variant. The p.Arg250Gln was observed in a VWS patient with bilateral lip pits and alveolar cleft only; however, no parental samples were available to determine whether this variant was de novo or segregated in the family. The p.Lys320Asn was observed in a VWS patient with bilateral CLP, bilateral lip pits and hypodontia; no parental samples were available. The splice acceptor site variant, c.175‐2A>C, was observed in NSOFC patient with left unilateral NSCLP, whereas the splice donor site variant, c.379 + 1 G>T, was found in a VWS patient with bilateral CLP and bilateral lip pits. These two variants are novel; however, some parental samples were not available for us to determine whether they were segregating or de novo.

**Figure 3:**
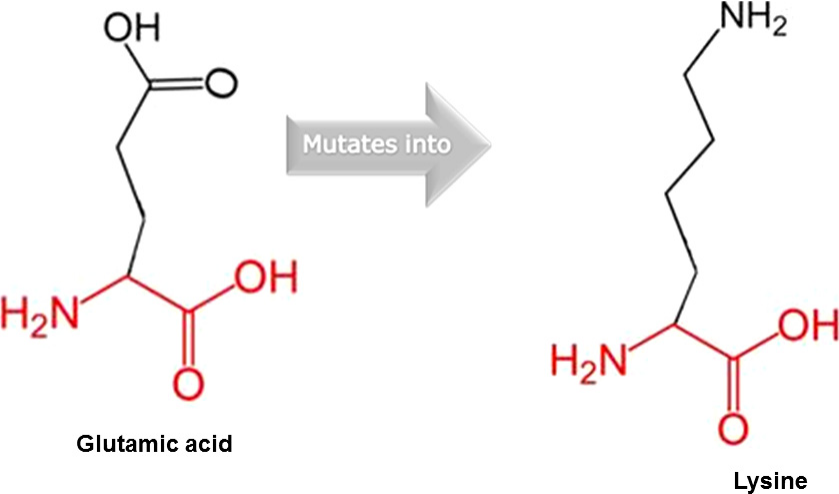
Substitution of Glutamate for Lysine in p.Glu69Lys mutant in *IRF6* (RefSeq NM_006147.3).

**Figure 4:**
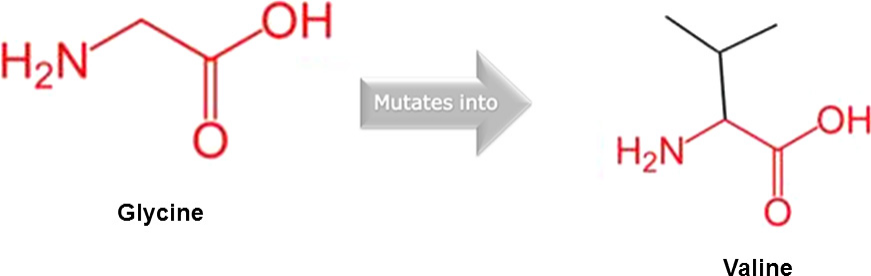
Wildtype and mutant amino acid residues for p.Gly65Val variant in *IRF6* (RefSeq NM_006147.3).

**Figure 5:**
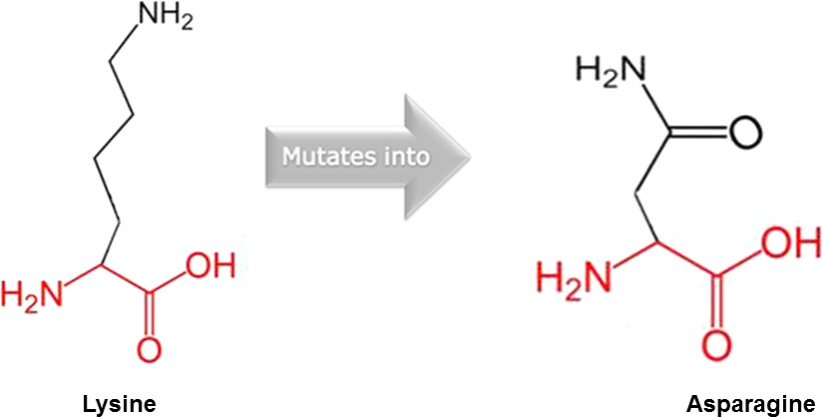
p.Lys320Asn wild‐type and mutant amino acid residues in *IRF6* (RefSeq NM_006147.3).

According to HOPE (http://www.cmbi.ru.nl/hope), three of the novel mutations affected the structure and function of *IRF6* protein for a number of biochemical reasons. Both the p.Glu69Lys and p.Gly65Val variants occurred in the highly conserved, N‐terminal helix‐turn‐helix DNA‐binding domain, whereas p.Lys320Asn occurred in the transactivation domain. For p.Glu69Lys, glutamate is relatively smaller and negatively charged whereas Lysine is relatively larger and positively‐charged (Fig. 3). The mutant valine residue in p.Gly65Val is larger and has relatively higher hydrophobicity than glycine (Fig. 4). For p.Lys320Asn variant, asparagine is relatively smaller and neutral in charge, whereas lysine is relatively larger and positively charged (Fig. 5).

## Discussion

We observed that etiologic variants in *IRF6* were nonrandomly distributed among the seven coding exons of the gene; we also could not detect *IRF6* etiologic variants in all VWS patients. All etiologic coding and splice site variants (12 out of 13 variants, about 92%), except p.Asn185Thr in exon 6, were distributed between exons 4 and 7 of the gene, confirming earlier observations (Kondo et al. 2002; Ferreira de Lima et al. 2009; Butali et al. 2014a). This observation is relevant for genetic testing in Africa: with limited resources, one can focus on exons 4 and 7 when the need arises to sequence the DNA of a subject. Moreover, we detected potentially etiologic *IRF6* variants in nine out of the 13 (about 69%) patients with VWS. The lack of etiologic *IRF6* variants in all VWS patients buttresses the point that other upstream or downstream *IRF6* target genes or genes in the same regulatory network as *IRF6* may also contribute to clefting in VWS and PPS patients, as etiologic *IRF6* variants are observed in only about 70% of VWS patients (Rorick et al. 2011; Wu‐Chou et al. 2013; Fakhouri et al. 2014; Peyrard‐Janvid et al. 2014; Kwa et al. 2015). Our analyses also showed that 18 out of the 184 patients with NSOFCs had potentially etiologic *IRF6* variants, suggesting etiologic *IRF6* variants occurred in about 9% of our study cohort. These 18 variants included both coding and splice site variants (a total of 12), as well as potentially etiologic regulatory region (enhancer) variants (a total of six). Furthermore, no etiologic *IRF6* variants were observed in other MCAs, save VWS patients. Interestingly, some of these MCA patients had phenotypes that have been reported in PPS patients (Ghassibe et al. 2004; Sarode et al. 2011]. These included hexadactyly, syndactyly, undescended testes, hypospadias, nail and toe aplasia, club foot, microphthalmia, etc. This observation suggests other known or unknown syndromes may account for these extracongenital anomalies, since over 300 syndromes present with cleft and other birth defects (OMIM).

We also observed that potentially etiologic variants in *IRF6* exhibited variable penetrance in that the lip pits that are usually associated with VWS were absent in the NSOFCs patients; some of these variants were also observed in both patients with overt cleft and clinically unaffected parents. Based on molecular evidence, the two patients with p.Glu69Lys variant are actually VWS patients, instead of NSOFC patients. However, during clinical assessment, the classical VWS phenotype that occurs with CL/P, lip pits, were absent. This suggests that though these a priori NSOFC patients are actually VWS patients, the lip pit phenotype was not penetrant, buttressing earlier observations on lack of lip pits or lip pit variability in individuals carrying etiologic variants in *IRF6* (Desmyter et al. 2010; Rutledge et al. 2010). The two patients that had the p.Glu69Lys variant had other variants that could have modified their phenotype: the NSCPO patient also had p.Pro408Leu variant in *PAX7* (OMIM:167410) whereas the NSCLP also had another mutation (p.Asn185Thr) in *IRF6*. Interactions between *IRF6* and other genes have been suggested by a study (Leslie et al. 2013). Therefore, compound heterozygosity and epistasis (gene‐gene interactions) may explain why phenotypes of *IRF6* etiologic variants are variable and why parents carrying single variants of either *IRF6* or *PAX7* had no overt cleft. It is also possible that parents who harbored potentially etiologic variant in *IRF6* or *PAX7* may also have subclinical phenotypes that have been associated with OFCs. These possibilities require further investigation in future studies. Thus, our study and other studies (Marazita et al. 2009; Dixon et al. 2011) presuppose the need to carry out deep subclinical phenotyping in families with history of clefts. This is warranted by the fact that some individuals without overt OFCs but that have certain subclinical phenotypes (such as orbicularis oris muscle defects, ankyloglossia, atypical lip pits or bumps, alterations in brain structure, facial asymmetry, velopharyngeal insufficiency, dental anomalies, congenital absence of uvula, etc.) have been shown to harbour etiologic *IRF6* variants (Nopoulos et al. 2007; Vieira et al. 2008; Neiswanger et al. 2009; Weinberg et al. 2009; Yeetong et al. 2009; Dixon et al. 2011).

Finally, we observed that patients carrying the same potentially etiologic variants in *IRF6* exhibited variable expressivity. The protein‐truncating variant p.Arg250X was observed in both nonsyndromic and VWS patients: the NSCL case had no lip pits whereas the VWS patients had lip pits. The two VWS patients with the p.Arg250X variant have variable phenotypes: a patient had bilateral CLP, ankyloglossia and bilateral lip pits whereas the other patient had right unilateral CL and bilateral lip pits. However, we could not get samples from all parents to determine whether this variant was de novo or segregated in these families. Variable VWS and PPS phenotypes which are usually caused by a single etiologic variant have been observed both within and between families (Ghassibe et al. 2004; de Medeiros et al. 2008; Ferreira de Lima et al. 2009; Birkeland et al. 2011; Malik et al. 2014). To make our observations very clinically relevant, future studies may aim at sequencing *IRF6* gene in larger cohort from Africa.

## Conclusion

We have shown for the first time in the Ghanaian population the evidence of novel etiologic *IRF6* variants in most patients with VWS as well as some NSOFC patients. In support of an earlier study, our study has shown that *IRF6* etiologic variants may be prevalent in the African population, however, with variable penetrance and expressivity. To the best of our knowledge, we have also shown for the first time an evidence of *IRF6‐IRF6* and *IRF6‐PAX7* compound heterozygotes that may modify phenotypes that emanate from *IRF6* etiologic variants. These observations are relevant for detection of high‐risk families, genetic counseling as well as future sequencing projects that may initially limits sequencing to exons 4 and 7 of *IRF6*. In conclusion, our study suggests there may be the need to combine both molecular and clinical evidence in the classification of OFCs into syndromic and nonsyndromic forms.

## Conflict of Interest

None to declare.

## Web Resources

www.omim.org; genetics@uiowa.edu; http://order.functionalbio.com/seq/index; http://www.umd.be/HSF/; http://www.1000genomes.org/; http://snp.gs.washington.edu/EVS/; http://genetics.bwh.harvard.edu/pph2/; http://sift.jcvi.org/; http://www.cmbi.ru.nl/hope

## Supporting information


**Table S1.** Phenotypes of patients with multiple congenital anomalies (MCAs).
