# Supplementary material for: The prevalence, penetrance, and expressivity of etiologic IRF6 variants in orofacial clefts patients from sub‐Saharan Africa
Source: Mol Genet Genomic Med. 2017 Jan 12;5(2):164–71. doi: 10.1002/mgg3.273 (PMC5370218; doi:10.1002/mgg3.273)
Supplement: Supplementary file 1 — Table S1. Phenotypes of patients with multiple congenital anomalies (MCAs). [file MGG3-5-164-s001.docx]

**Table S1**: Phenotypes of patients with multiple congenital anomalies (MCAs)

| Syndromic forms of OFCs | Total |
| --- | --- |
| Pierre Robin Sequence (PRS) | 9 |
| Van der Woude Syndrome (VWS) | 13 |
| Cleft-club foot only | 6 |
| Goldenhar Syndrome | 5 |
| Amniotic Band Syndrome | 3 |
| Mobius Syndrome | 2 |
| DiGeorge Syndrome | 1 |
| Fragile-X Syndrome | 1 |
| Edward Syndrome | 1 |
| Opitz Syndrome | 1 |
| Apert Syndrome | 1 |
| Stickler Syndrome | 1 |
| Cri du Chat Syndrome | 1 |
| Kabuki Syndrome | 1 |
| Holoprosencephalon | 1 |
| Other multiple congenital anomalies (MCAs) | 33 |
| Total | 80 |

Twenty-five of the MCAs presented with a spectrum of abnormalities that included overt OFCs, hexadactyly, microphthalmia, anophthalmia, microform clefts, ankyloglossia, talipes equinovarus (club foot), penoscrotal webbing, undescended testes, hypospadias, ocular hypertelorism, wide low-set ears, malformed ear lobe and coloboma. This spectrum of phenotypes is characteristic of pterygial syndromes, such as popliteal pterygium syndrome, PPS (OMIM). Three other probands had clefts with other anomalies such as frontonasal dysplasia, microcephaly, developmental delays, toe aplasia and hypoplasia as well as hyperdontia. Moreover, cleft that present with hole-in-heart, stunted growth, motor dysfunction, deafness, dumbness, bent or defective vertebral column as well as seizures was observed in a proband. Two other individuals also had clefts that presented with a number of developmental delays: severe mental retardation, learning disability, language disability, other global developmental delays, microcephaly, ocular hypertelorism, low-set ears as well as hearing and visual impairments. A proband also had cleft together with imperforate anus, which led to faecal discharge through the vagina. Finally, an individual also had cleft that presented with ptosis and proptosis of eyes, club foot as well as choanal atresia.
